# Internet of Things Platform for Smart Farming: Experiences and Lessons Learnt

**DOI:** 10.3390/s16111884

**Published:** 2016-11-09

**Authors:** Prem Prakash Jayaraman, Ali Yavari, Dimitrios Georgakopoulos, Ahsan Morshed, Arkady Zaslavsky

**Affiliations:** 1 Department of Computer Science and Software Engineering, Swinburne University of Technology, Melbourne 3022, Australia; dgeorgakopoulos@swin.edu.au (D.G.); amorshed@swin.edu.au (A.M.); 2 Data 61, CSIRO, Melbourne 3168, Australia; ali.yavari@rmit.edu.au (A.Y.); Arkady.Zaslavsky@csiro.au (A.Z.); 3 Computer Science and Information Technology, RMIT University, Melbourne 3001, Australia

**Keywords:** Internet of Things, smart agriculture, semantic web

## Abstract

Improving farm productivity is essential for increasing farm profitability and meeting the rapidly growing demand for food that is fuelled by rapid population growth across the world. Farm productivity can be increased by understanding and forecasting crop performance in a variety of environmental conditions. Crop recommendation is currently based on data collected in field-based agricultural studies that capture crop performance under a variety of conditions (e.g., soil quality and environmental conditions). However, crop performance data collection is currently slow, as such crop studies are often undertaken in remote and distributed locations, and such data are typically collected manually. Furthermore, the quality of manually collected crop performance data is very low, because it does not take into account earlier conditions that have not been observed by the human operators but is essential to filter out collected data that will lead to invalid conclusions (e.g., solar radiation readings in the afternoon after even a short rain or overcast in the morning are invalid, and should not be used in assessing crop performance). Emerging Internet of Things (IoT) technologies, such as IoT devices (e.g., wireless sensor networks, network-connected weather stations, cameras, and smart phones) can be used to collate vast amount of environmental and crop performance data, ranging from time series data from sensors, to spatial data from cameras, to human observations collected and recorded via mobile smart phone applications. Such data can then be analysed to filter out invalid data and compute personalised crop recommendations for any specific farm. In this paper, we present the design of SmartFarmNet, an IoT-based platform that can automate the collection of environmental, soil, fertilisation, and irrigation data; automatically correlate such data and filter-out invalid data from the perspective of assessing crop performance; and compute crop forecasts and personalised crop recommendations for any particular farm. SmartFarmNet can integrate virtually any IoT device, including commercially available sensors, cameras, weather stations, etc., and store their data in the cloud for performance analysis and recommendations. An evaluation of the SmartFarmNet platform and our experiences and lessons learnt in developing this system concludes the paper. SmartFarmNet is the first and currently largest system in the world (in terms of the number of sensors attached, crops assessed, and users it supports) that provides crop performance analysis and recommendations.

## 1. Introduction

Improving farm productivity requires crop performance to be understood and forecasted under a wide variety of environmental, soil, fertilisation, and irrigation conditions. Productivity of a farm can be enhanced by determining which crop variety has produced the greatest yield under similar soil, climate, fertilisation, and irrigation conditions. The same data-driven approach to crop selection can also address climate change, resource constraints (water, labour, and energy shortages), and societal concerns around issues such as animal welfare, fertilizers, and environment that often impact agricultural production [1]. According to the United Nations’ Food and Agriculture Organization [2], food production must increase by 60% by 2050 to be able to feed the growing population, expected to reach 9 billion. Increased crop productivity is urgently needed, and it is the cornerstone of any solution for meeting food shortage and farm profitability problems. Smart farming involves the use of Information Communication Technologies (ICT) and in particular, the Internet of Things (IoT) and related big data analytics to address these challenges via the electronic monitoring of crops, as well as related environmental, soil, fertilisation, and irrigation conditions. Such monitoring data can be then be analysed to identify which crops and specific crop varieties can best meet the productivity targets of any particular farm around the world. Crop variety identification involves the use of plant phenomics (an area of biology concerned with the measurement of phenomes—the physical and biochemical traits of organisms—as they change in response to genetic mutation and environmental influences [3]). Therefore, smart farming permits the assoication of crop data (i.e., crop performance, environmental, soil, irrigation, and fertilisation data) and related data analysis results with specific crop varieties (i.e., plant genes and phenotypes). The association of information will revolutionize the way food is produced globally.

To observe the growth of the crop under varying real-world conditions (e.g., soil quality, environmental conditions, etc.), typical crop studies involve phenotyping to understand the key factors (e.g., the pH levels of soil, the rate of Nitrogen depletion) affecting growth. Such studies are conducted in natural outdoor environmental conditions and locations where plants are growing, by varying irrigation and the application of fertilizers/additives. Internet of Things (IoT) technologies can lower the cost and increase the scale of such studies via the collection of related time series data from sensor networks, spatial data from imaging sensors, and human observations recorded via mobile smart phone applications [4]. For example, IoT devices can help to capture the pH levels of soils and the rate of Nitrogen depletion as time-series data, and share it among interested researchers and growers for further analysis.

Point solutions for smart farming currently exist , but they can only utilise a small number of specific IoT devices (e.g., a specific model of soil humidity sensor), and provide no support for data analysis or sharing. Using such existing solutions also requires a significant effort in order to integrate and correlate the data obtained from different IoT devices , e.g., data from a fertilizer sprayer on a tractor (made by one manufacturer) with the data obtained from soil moisture sensors (made by a different manufacturer). Existing solutions are not designed around a bring-your-own IoT sensor principle that will allow the use of new IoT devices without modification, and permit such solutions to keep up with the rapid development of cheaper and better IoT sensors. Furthermore, none of the existing solutions are designed for comprehensive and scalable analysis, recommendation/visualisation, or sharing of crop performance data among farmers, growers, biologists, government, and commercial organisations that support farming operations and produce relevant products.

In this paper, we present SmartFarmNet, an IoT platform for smart farming applications that provides the following:

1. Allows a bring-your-own IoT sensor principle , i.e., permits effortless integration and use of virtually any IoT device, including commercially available sensors, cameras, weather stations, etc. This reduces sensor installation and maintenance costs, while providing for easy upgrade to newer and more advanced sensors.
2. Supports scalable data analytics that can continuously process large crop performance data.
3. Offers do-it-yourself tools that allow plant biologists and farmers/growers to analyze and visualize plant performance data.

SmartFarmNet was developed by a multi-disciplinary Australian team that included crop biologists, computer scientists, growers, and farmers. SmartFarmNet is largest system in the world (in terms of the number of sensors attached, crops assessed, and users it supports) that provides crop performance analysis and recommendations. It is also the first system to provide the features (1)–(3) above, which are the main innovations presented in this paper. Moreover, SmartFarmNet provides tools for fast and scalable data that can cope with the enormous velocity of data (Big Data) generated from hundreds of thousands of IoT sensors.

The rest of the paper is organised as follows. In Section 2, we present a smart farming use case. Section 3 presents the current state-of-the-art, and discusses the challenges in developing IoT-based solutions for smart farming. Section 4 presents the architecture of the SmartFarmNet platform and highlights its key features. Section 5 covers the software implementation of the platform. Section 6 presents evaluation results, while Section 7 outlines lessons learned. Section 8 concludes the paper.

## 2. A Use Case and Related Challenges for Smart Farming

Phenonet [5] is an agricultural phenotyping field laboratory, involving a variety of crop studies that are being conducted using state-of-the-art IoT technologies [6], including sensor networks, IP cameras, mobile smartphones, and related data analytics. These enable near real-time capture of crop data for assessing and predicting crop performance (both short-term and long-term) for any given environmental, soil, fertilisation, and irrigation conditions, including weather conditions, plant canopy temperature, soil moisture, soil quality and salinity, fertiliser usage, and irrigation. Phenonet is helping plant biologist and growers achieve the following: (1) identify the influence of different conditions on a variety of crops in real-world outdoor farm environments; (2) understand water resource consumption in order to manage it effectively; (3) study the impact of various fertilizers; (4) get real-time data to forecast crop performance; and (5) share data and results. To better explain Phenonet, we provide additional details about sample studies conducted in Phenonet. One of these studies aim to evaluate the effect of sheep grazing on crop re-growth by looking at root activity, water use, rate of crop growth, and crop yield. In this study, soil moisture sensors deployed at multiple depths and canopy temperature sensors are used to track the crop roots’ extraction of water from the soil throughout the growing season. This information is then used to measure and assess root activity and crop growth.

**Figure 1:**
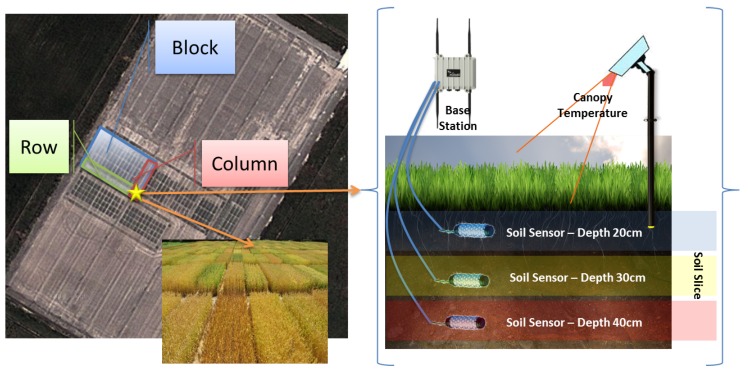
Overview of Phenonet.

Figure 1 presents an overview of this Phenonet study that is conducted on a block of land that is divided into hundreds of plot. A plot is an intersection of a row and a column in a block of land and hosts a specific treatment to the crop being studied. For example, each plot may host a different application of fertilizers or host the application of the same fertilizer at different times. This Phenonet study requires support for real-time collection and delivery of data to biologists, who then share their insights with growers and farmers, as well as related scientific organisations. Some of the main challenges in supporting such studies and also normal farming activities include the following:

- Capturing the large volume of heterogeneous data produced by a variety of IoT sensors (and possibly manual measurements), and doing this for a large number and variety of activities involving different studies as well as crops.
- Supporting the integration and use of almost any IoT device, including all commercially available sensors, camera, weather stations, etc., as this will achieve a bring-your-own sensor model of operations that will allow farmers, growers, and scientists to take advantage of cheaper/more capable IoT sensors, as well as individual preferences and budgets.
- Integrating heterogeneous data from such a great variety of IoT devices and also historical crop performance data produced by past studies (such data and results are typically available in CSV files that make it harder to use, analyse, explore, and share).
- Providing crop performance data analysis software and related tools for do-it-yourself search, analysis and visualisation of collected data across multiple studies.
- Sharing sensors, data, analysis tools, results, and data visualizations.

## 3. Related Work and Challenges is Building IoT-Based Platforms for Smart Farming

Relational database systems (e.g., mysql [7]) have been the historic choice for storing data from the sensors for later retrieval, analysis, and visualisation. However, the performance of these systems can be compromised by large streams of time-series data, and they do not address most of the challenges outlined in the Section 2.

To meet such challenges vendors such as John Deere [8], IBM [9], and SensorCloud [10] currently explore or provide proprietary solutions that support specific sensors. However, vendor-specific solutions provide limited or no interoperability with other devices in the IoT ecosystem. Vendor lock-in is also a serious issue.

A plethora of IoT middleware platforms has recently appeared in the marketplace. Examples include UBIDOTS [11], Xively [12], Thing Speak [13] Open.Sen.se [14], and SensorCloud [10] and more recently, Amazon IoT [15] and IBM IoT [16] platforms. These IoT middleware platforms aim to simplify the injection of data from all kinds of sources (physical devices, human input, online data, etc.) using a common Application Programming Interface (API) . All such platforms typically provide basic functionalities for filtering and aggregating the data, as well as specification of events based on input data. Although the IoT platforms in this category (e.g., those based on IBM (Bluemix) and Amazon (EC2)) provide tools for data ingestion, storage, and computation via message passing interfaces utilising message queues and publish/subscribe mechanisms, the benefits of these IoT platforms in smart agriculture is yet to be determined. In contrast to such lack of track record in this area, the SmartFarmNet platform we introduce in this paper (as well as its earlier variants and components) has been deployed across Australia and it has been successfully used in collecting and analysing data from tens of thousands of sensors deployed in remote field locations. Furthermore, the outcomes presented in this paper are results and experiences gained obtained from four years of real-world deployments.

The category of open source IoT platforms includes the IoTCloud architecture [17] project that originated from the Community Grids laboratory in Indiana University, USA. The IoTCloud architecture provides APIs for sensors to publish data to the cloud and for clients to subscribe to such data. IoTCloud takes advantage of a publish–subscribe architecture to support scalable ingestion and querying of sensor data. However, it lacks bring-your-own support for IoT devices, and does not provide fast, scalable analysis of sensor data. Other platforms in this category include the Data Turbine [18] and Apache Storm [19]. These IoT platforms focus only on the data ingestion layer. In particular, these middleware systems act as a black box through which applications and devices exchange data. The SmartFarmNet platform provides a bring-you-own IoT device integration capability that includes virtually any IoT device that may be provided by any user (e.g., farmer, grower, or scientist). In particular, SmartFarmNet currently provides more than 30 wrappers for most commercial (e.g., libelium [20] and experimental (e.g., arduino [21], tMotes [22], etc.) sensor platforms.

IoT platforms that can utilise IoT devices provided independently by a third party must provide for sensor discovery [23] (i.e., ability to search sensor metadata). Although there has been extensive work in providing metadata management for sensory data [24,25], no IoT platform currently fully supports sensor discovery. For instance, projects such as SensorMap [26] have visualised live sensor data on a map, allowing users to find sensors based on the sensor location. SmartFarmNet takes advantage of semantic web principles (such as Linked Open Data and Semantic Sensor Ontology (SSN) [27]) to search for and share sensor information in a way that can be read automatically by computers.

Existing IoT-platforms are not designed to support near-real-time data ingestion, quick analysis, and visualisation of large volumes of sensor data. One of the key features of the SmartFarmNet platform is in its ability to deal with the high velocity of sensor data (time-series) by providing solutions for fast and scalable data analytics and retrieval. The do-it-yourself principles-driven user-interface allows complex data workflows to be built, composed, and executed without a need for programming. SmartFarmNet also provides a web-based virtual laboratory environment for biologists, farmers, and scientists to manage large volumes of crop sensor data while supporting rapid responses to queries, real-time user interaction, and the ability to share data from studies performed by multiple researchers. A comparative analysis of current IoT platforms and the proposed SmartFarmNet platform is presented in Table 1.

## 4. The SmartFarmNet IoT Platform for Smart Farming

### 4.1. SmartFarmNet’s Data Model

**Figure 2:**
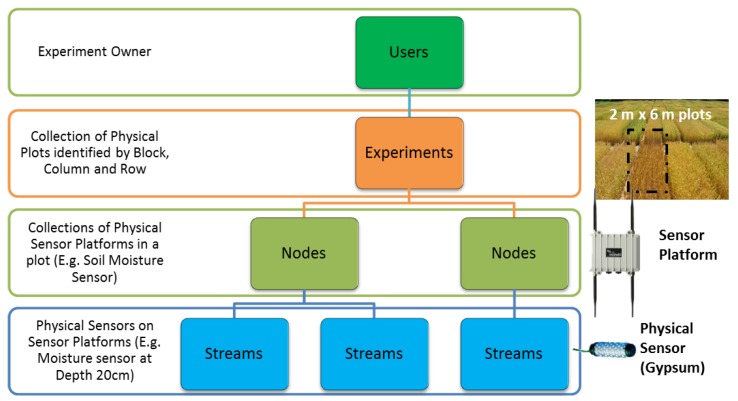
SmartFarmNet’s data model.

The data model used by the SmartFarmNet platform is depicted in Figure 2. A user is a logical entity representing a project, a research group, or any other entity that owns data from a study. Experiments are crop studies, and each experiment has only one owner user. Tests are comprised of a collection of nodes, but each node can belong to multiple crop studies. A node can also have a location with it, such as latitude and longitude. A node is a group of data streams (from virtually any IoT device or even virtual sensors, e.g., CSV files from past studies). A stream is a time series data from an experiment with a unit of measurement. Metadata can be attached to every hierarchical layer of the data model. The following policies are enforced by the data model.

- Any user can have zero or more studies.
- Any study can have zero or more nodes.
- Any node can have zero or more streams. Each node can also have latitude, longitude, and altitude values.
- Any stream is a set of (timestamp, value) pairs. Each stream has one unit of measurement.

The layout of a typical crop study (referred to as “experiment” in the data model) is illustrated in Figure 2. Such studies involve a particular area (one or more plots) in a field. Any farm can be divided into plots, and the granularity of the analysis supported by SmartFarmNet is currently 9–10 square meters. The plots are mapped to the node level that represents the sensor network platform (hosting all related communication and processing capabilities). Physical sensors observing a physical phenomenon (e.g., gypsum block sensors measuring soil moisture at multiple depths) are connected to corresponding streams. A measurement of soil moisture at a particular depth is mapped to a data stream associated with a node. In summary, the data streams map to the physical or virtual sensors that monitor a phenomenon, while nodes and experiments/studies are used for grouping streams at different levels. The metadata associated with nodes and streams at each level are critical for providing contextual information. In Phenonet, metadata at the experiment/study level may include the year when the study was conducted, the date the crop was sown, a description of the objectives of the study, and even descriptions about study site (e.g., the soil type). At the node level, the most important metadata fields are the plants’ genotype and its relative location within the plot (in most cases, a row/column notation is used). Treatments applied to individual plants can also be appended as metadata at the node level. The most relevant metadata at the stream level for Phenonet are the depth, the sensor type, and the sensor serial number, while sensor information such as the date of calibration or setting of the sensor can also be critical.

### 4.2. Bring-Your-Own Sensors Support via the Semantic Sensor Network Ontology

Providing bring-your-own sensors support involves the use of Semantic Sensor Network (SSN) ontology to represent the underlying IoT devices and the data generated by such devices. The SSN ontology was developed by World Wide Web Consortium (W3C) Semantic Sensor Networks Incubator Group [27], and provides a schema that describes sensors, observation, data attributes, and other related concepts. The SSN ontology is widely used, and has been utilised in a significant number of Semantic Web and IoT projects, including SmartFarmNet. Linked Data is also used to relate and bind SSN concepts across multiple sensor platforms. The four main principles [28] of linked data include: (1) using unique Uniform Resource Identifier (URIs) to represent each IoT thing; (2) providing HTTP interfaces to access the URIs (descriptions); (3) offering information related to URIs via the Resource Description Framework (RDF) [29]; and (4) linking URIs. The linked data approach allows sensor descriptions and sensor data to be linked with corresponding domain-specific knowledge.

**Figure 3:**
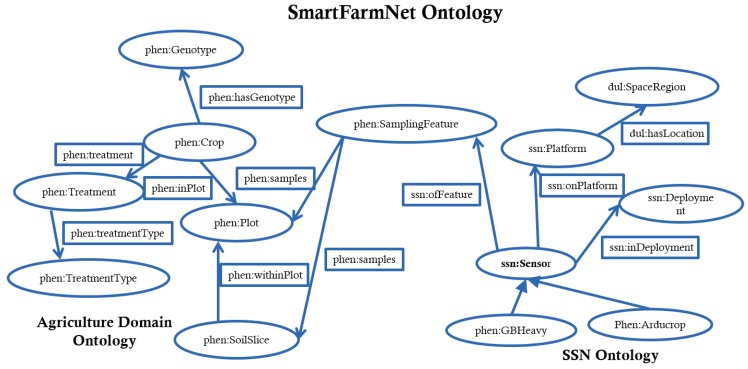
The SmartFarmNet ontology (phen denotes the phenonet ontology namespace, dul denotes the DOLCE+DnS Ultralite ontology namespace and ssn denotes the SSN namespace).

The SmartFarmNet ontology (Figure 3) is an extended version of the SSN ontology that describes the SmartFarmNet data model we presented in Section 4.1. The ontology provides the means to map the data model to sensor descriptions. For example, a soil moisture sensor *X* is deployed in a plot *Y*, which grows a crop *Z*. Sensor *X* and its data are described using the SSN ontology. This ontology includes the physical and technical nature of the installed sensor network, such as what physical platform the sensor is on, and location of the sensor. The description of the plot *Y* and crop *Z* is based on the Descriptive Ontology for Linguistic and Cognitive Engineering (DOLCE) Ultra-Light upper ontology [30].

The SmartFarmNet Ontology describes the plots, the crops and the corresponding treatments applied to the crops. For example, consider crops with a particular genotype (e.g., revenue) that are sown in plots belonging to a study. This crop is subjected to different treatment (e.g., irrigation). The sensors deployed in the plots are governed by a geo-location observation system either at the plot level, or at the level of some sub-feature of the plot (e.g., a layer of soil within the plot), or as the entire site of the study (e.g., temperature and humidity covering the whole study site). The sampling feature links the sensors to corresponding plots, and from the plot to the combination of genotype, treatments, and events (such as sowing dates, etc). Features can be related such as a soil layer can be from an individual plot or from several plots linked together into a study site. The ability to connect features means that measurements of larger phenomena (such as the site weather) can be associated to individual plots and plants. The ontology allows data to be mapped to the semantics of the SmartFarmNet, enabling on-the-fly automatic annotation of sensor data streams.

### 4.3. Scalable Data Analysis of Sensor Data in SmartFarmNet

SmartFarmNet provides near real-time response to queries over time-series data streams from sensors. Just like in the SensorDB [4] platform we developed earlier, the SmartFarmNet platform uses an internal data structure called micro summarisation to build a summary of statistical features for incoming sensor data streams. Furthermore, SmartFarmNet internally uses aggregation windows. There are several collection windows defined, including 1-min window, 15-min window, 1-h window, 1-day window, 1-month window, 1-year window, and a start-of-time window. Each aggregation window maintains the following information about the data points it holds: the number of data points in each window, the minimum and maximum values, and the average and standard deviation of the data points inside the window.

**Figure 4:**
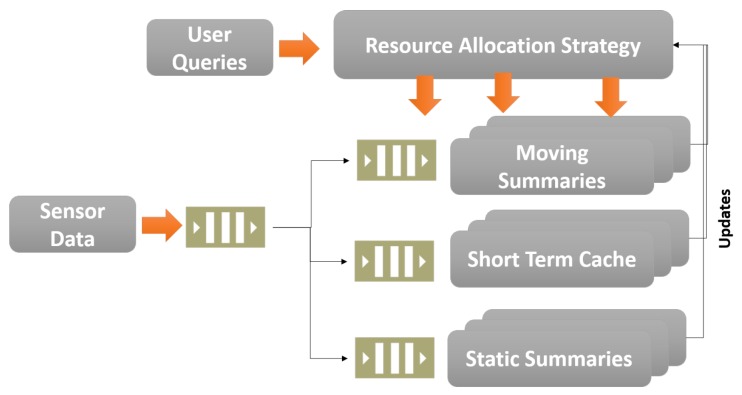
SmartFarmNet—scalable data analysis of sensor data.

SmartFarmNet uses a combination of non SQL (NoSQL) and Semantic data stores to manage user data, sensor data, aggregated data, and caching of commonly used information. The storage layer behind each aggregation window is allocated based on query access patterns. Using this approach, we can optimize micro-summary computations for different scenarios. For instance, if a data item at the 1-h aggregation window is accessed more frequently than the 1-min aggregation window, SmartFarmNet can be configured to store and cache data in that collection window in faster storage, such as in memory storage or a solid state disk drive. The data stream processing model of SmartFarmNet is presented in Figure 4. The platform provides three levels of data storage: (1) Short Term Cache; (2) Moving Summaries; and (3) Static Summaries. The summaries computed over the moving aggregate window are termed “moving summaries”, and are updated with new incoming data streams. The static summaries are provided for data streams whose data does not change rapidly over time, hence not requiring frequent re-computation of summaries. Finally, the short-term cache is used to store raw data in order to improve the response time of raw data requests. The resource allocation strategy uses the combination of the query and the performance of each storage to determine where to store incoming data streams. The unique feature of the platform is its elasticity. A stateless process thread works on the queue (at the left of Figure 4) to process each individual sensor data stream. Therefore, we can achieve a high degree of distributed and parallel processing that can utilise available computing resources very efficiently. The stream maintains the stream identifiers that link it to the moving micro-summaries of all related streams computed over days, weeks, months, and years.

### 4.4. SmartFarmNet Architecture

**Figure 5:**
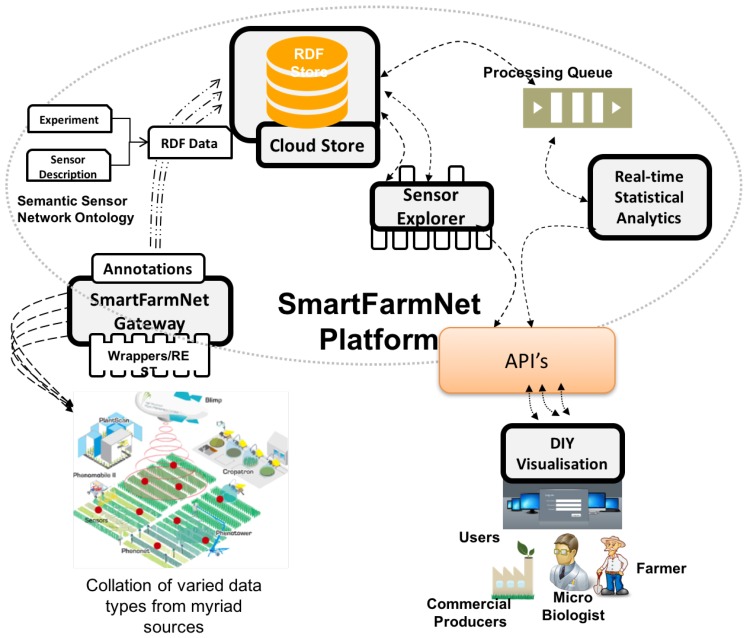
SmartFarmNet architecture. DIY: do-it-yourself; RDF: resource description framework; API: Application programming interface.

Figure 5 depicts the architecture of the SmartFarmNet platform. SmartFarmNet provides support for bring-your-own sensor, i.e., the ability to integrate and describe virtually any IoT device, including sensors, mobile smart phones, cameras, farming equipment, etc. The data generated from such IoT devices are initially processed on local SmartFarmNet gateways. The SmartFarmNet gateways communicate with both the sensors and the SmartFarmNet Platform running in the cloud. The SmartFarmNet gateways also allow processing of sensor data closer to the source, enabling improved usage of communication bandwidth between remote sites and the cloud platform. The SmartFarmNet platform on the cloud is responsible for storage and performing real-time analysis on incoming sensor data streams. It also provides do-it-yourself-driven interfaces for user queries, and for interactive visualisation and sharing of sensor data and analysis results. The distribution model used by SmartFarmNet enables sensor data collected in one study to be used in other studies (e.g., temperature data gathered from a crop study can be re-purposed to study the movement of locust, based on weather conditions). In the following itemized list we provide a description of various components of the SmartFarmNet platform specifically designed and developed to address the challenges identified in Section 2.

- *SmartFarmNet gateway*: This collects, filters, and collates data streams from virtually any IoT device. The SmartFarmNet gateway uses the OpenIoT X-GSN component [31] for data ingestion. The component communicates with sensors using wrappers. A wrapper is an interface that allows the gateway to pull/push data from/to the underlying IoT device. The SmartFarmNet platform currently has inbuilt support for data ingestion from more than thirty IoT device platforms, including Arduino, Netatmo, Libelium WaspMotes, Remote, IP-Cameras (to name a few). It also provides support for virtual sensors such as CSV files. The data from the IoT devices are annotated on-the-fly with metadata describing the IoT device and the data is encoded using the SmartFarmNet ontology described in Section 4.2. In Section 5, we will introduce the sensor schema editor that is used to describe all employed sensors via a graphical user interface. This graphical interface hides the complexities of dealing with ontologies from the user, and also provides support for the uploading of historical data. The gateway can be deployed across multiple sensor sites/locations, and data could potentially be aggregated at an intermediate location (via SmartFarmNet gateways) or in the cloud. The annotated data from the IoT devices are represented using the resource description framework (RDF) and stored in the cloud store. A sample RDF stream computed from the description of a sensor is presented in the listing below. This listing involves a Canopy Temperature sensor deployed in plot *4001*, which is part of the study *kirkegaard-and-danish*.


		<rdf : Description rdf:about="http://sensordb.csiro.au/phenonet/sensor/
		arducrop/20140611–1962–0012">
		<ssn : onPlatform rdf:resource="http://sensordb.csiro.au/
		phenonet/experiment/kirkegaard-and-danish/plot/4001
		/platform/phen077"/>
		<ssn:inDeployment rdf:resource="http://sensordb.
		csiro . au/phenonet/deployment/site/ges–creek–range/20140611-1962-0000"/>
		<ssn : ofFeature rdf:resource="http://sensordb . csiro . au/phenonet/experiment
		/kirkegaard–and–danish/plot/4001/sf"/>
		<rdfs : label rdf:datatype="http://www
		.w3.org/2001/XMLSchema#string">Canopy Temp</rdfs:label>
		<rdf : type rdf:
		resource="http://sensordb . csiro . au/ontology/phenonet#ArduCrop"/>
		</rdf : Description >

- *Cloud Store*: This enables the storage and management of data streams generated by the SmartFarmNet gateway. The cloud store uses the linked sensor middleware-light (LSM-Light) component of OpenIoT [31]. The cloud infrastructure stores all of the relevant sensor annotations (descriptions and metadata), the ontology, functional data related to user accounts, and permissions to enforce privacy and security. SmartFarmNet uses a semantic data store (a No-SQL graph database) to store the sensor data in RDF format. It internally implements publish/subscribe queues to handle large streams of sensor data stemming from virtual and physical sensors. The components provide APIs to perform the basic create, read, update and delete (CRUD) operations over the sensor data, and is responsible for transforming the data generated by the IoT devices into RDF triples using the SmartFarmNet ontologies.
- *Sensor Explorer*: This responsible for sensor discovery function, a novel feature of the SmartFarmNet platform. The detection feature uses ontology matching, allowing data collected to be re-used/re-purposed (i.e., re-purpose data gathered from one study for another study). For example, using the SmartFarmNet ontology, a semantic reasoner can discover data sources (IoT devices) that measure canopy temperature data deployed in plots that have had a specific nitrogen fertilizer treatment applied (study property). The discovery process provides much more features than a simple filtering operation by using semantic matching and reasoning methods. The sensor explorer uses the Scheduler and Service Delivery and Utility Manager of OpenIoT [31] for sensor and data discovery.

## 5. SmartFarmNet Platform Implementation

As mentioned before, the SmartFarmNet platform is built on the widely used open source platform for Internet of Things, namely OpenIoT [31], available for free download [31]. Table 2 presents the implementation details of the platform. The entire platform is deployed in a JBOSS [32] application container, while the support for real-time scalable data analytics is built independently using Redis, an in-memory data store.

### 5.1. Do-It-Yourself User Interfaces

#### 5.1.1. Sensor Schema Editor

**Figure 6:**
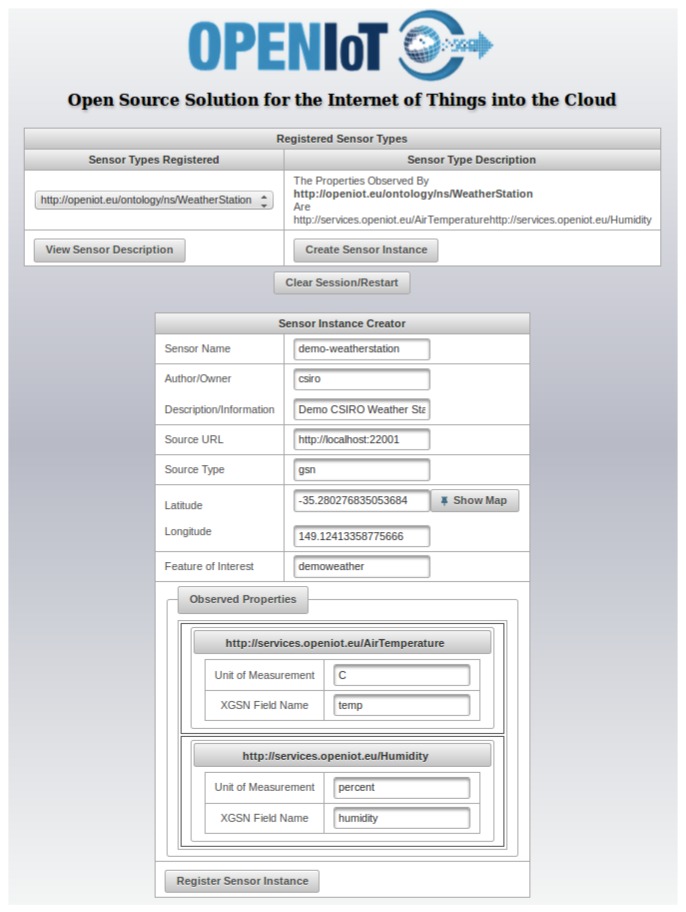
Sensor Schema Editor (SSE).

SmartFarmNet is designed with the Bring-Your-Own sensor principle and supports virtually any IoT provided by any vendor. The Sensor Schema Editor (SSE) enables users to describe and register a sensor with ease and consistency. This process hides the complexities of editing OWL files (used to represent ontologies) from the users. The Sensor Schema Editor provides annotations to sensors and sensor-related data, using the underlying ontology and Linked Data principles. The interface automates the generation of RDF descriptions for sensor node information submitted by the users. Internally, the editor performs dynamic extension of the ontology by making the newly-added concepts readily available via the user interface. Figure 6 presents a screenshot of the sensor schema editor used to define a sensor.

#### 5.1.2. Sensor Data Visualisation

**Figure 7:**
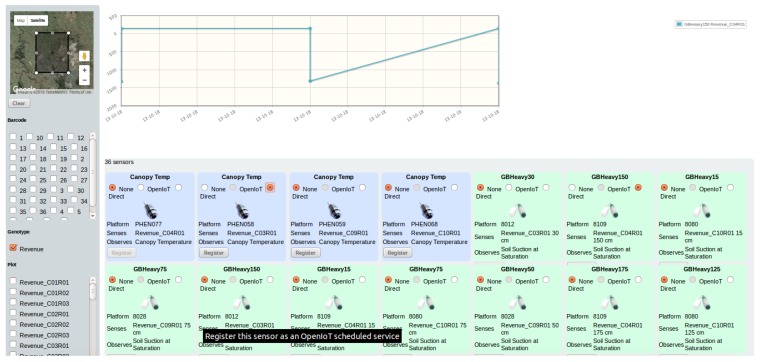
Sensor data discovery and exploration.

The SmartFarmNet platform user interface (UI) allows end-users to explore and analyse agricultural study data with zero-programming efforts. The UI hides the complexities of semantics and ontological representations from the user by presenting concepts that the user is familiar with and understands (e.g., plots, studies, sensor device, barcode, etc.), as defined in the data model and the ontology. The UI is depicted in Figure 7, and has the following features:

1. *Resource Discovery:* The user interface allows a user to search for sensors based on domain-based criteria, including genotype, crop treatment, and the barcodes used to identify studies, as well as sensor-based criteria, such as specific platform or location dynamically obtained from the ontology (discovery based on location and genotype i.e. Revenue is presented in the left side of Figure 7).
2. *Query Composition:* The provide search interface presents a list of sensors matching the discovery criteria provided by a user (depicted in the bottom part of Figure 7). The user can then compose a query targeted to the selected sensors. Internally, this makes a request to the Sensor Explorer and the real-time statistical analysis components.
3. *Service Visualisation:* This visualizes the fetched data using a one of the provided visualisation outputs, such as the time-series graph illustrated in the top part of Figure 7.

## 6. SmartFarmNet Evaluation

An essential design requirement of the SmartFarmNet platform is to be able to scale, store, process, and several hundreds and thousands of IoT sensors. The volume of data generated by the IoT sensor is not an issue. However, the velocity at which the data is produced is very high and results in billions of sensor data points. The objective of this evaluation is to evaluate the scalability of the SmartFarmNet platform. In particular, we evaluate the proposed real-time statistical analysis feature of the SmartFarmNet platform used to deliver sub-second query response latency while handling high-velocity data streams.

For evaluating the scalability of the platform, we used real data collected from a Phenonet field study called "Kirkegaard and Danish". This study evaluates the effect of sheep grazing on crop re-growth by looking at root activity, water use, crop growth rate, and crop yield. We used soil moisture sensors deployed at multiple depths (e.g., from 10 cm to 2 m) below the soil surface. These sensors provide a high-dimensional view of the extraction of water from the soil by the roots throughout the crop growing season. This information is then used to obtain an indirect measurement of root activity, which in turn can be used to control crop growth to increase yield and reduce fertiliser and water usage. The data for the studies was collected in real-time from 50 soil-moisture sensors, resulting in 50 streams of around 10,000 data points each.

SmartFarmNet platform uses SPARQL—a semantic query language similar to SQL —to query the sensor data. A sample (partial) SPARQL query is presented below. This query retrieves all data from sensors that measure soil moisture defined as *<http://purl.oclc.org/NET/ssnxlcf/cfproperty#soil_suction_aCsaturation>*. The FILTER statement of SPARQL is used to restrict the results from those sensors that are in the plot covered by the Kirkegaard and Danish study. The SPARQL query is an example of how semantic web concepts used effectively to query not only over the data (soil moisture sensor), but also the domain knowledge (studies, plots, etc.). The server used to evaluate the performance of our platform is an Amazon Elastic Cloud Computing (EC2) infrastructure [34] with the following hardware configuration: 8GB RAM, 2 vCPU (Intel(R) Xeon(R) CPU E5-2676 v3 @ 2.40GHz).


	  select ?sensor, ?values from <graphname> where {
	  ?sensor ssn:type ?type .
	  ?type ssn:observes  <http://purl.oclc.orgINET/ssnxlcf/cfproperty#
	  soil_suction aCsaturation>
	  ?sensor ssn:ofFeature ?samples
	  ?sensor ssn:Observation ?observation
	  ?observation ssn:ObservationValue ?values
	  ?samples phenonet:samples ?plot
	  ?crop phenonet : treated/phenonet : treatmentType
	  ?treatment
	  FILTER ( bound(?treatment ) && ( ?treatment =
	  <http://sensordb.csiro.au/id/treatment_type/grazed_high_n>))
	  }
	  

### 6.1. Query Access Performance Latency

**Figure 8:**
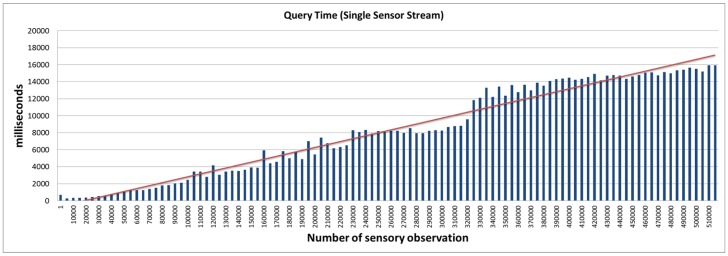
Query access latency—single sensor stream.

**Figure 9:**
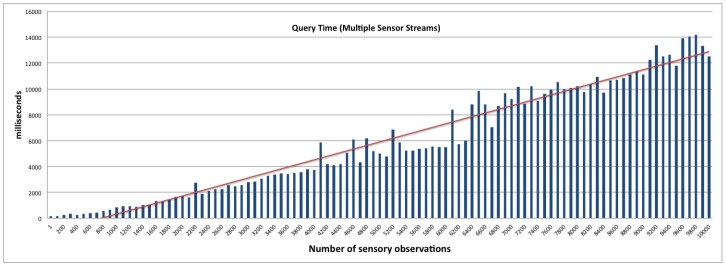
Query access latency—multiple sensor stream.

**Figure 10:**
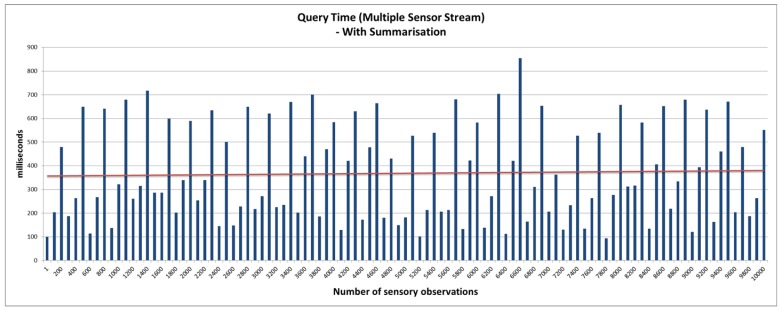
Query access latency with real-time statistical analysis—multiple sensor stream.

The query access performance latency is a good indicator of the system’s ability to support a high-velocity data stream while delivering sub-second query response. This is achieved by the real-time statistical analysis feature that uses the micro summarisation approach (presented in Section 4.3 ). First, we establish a baseline performance of the platform without using the real-time statistical analysis feature. We then compare the outcome of the benchmark against evaluations conducted using the real-time statistical analysis feature. The real-time statistical analysis feature allows the platform to respond to user queries by looking for pre-computed statistical data from the various smart summarisation data stores (cache, static, and moving). Depending on the frequency of the query, the platform makes a decision to move micro summaries from one storage to another. This feature is currently implemented via a configuration file. However, in the future, we aim to extend the platform with a smart caching approach that can make autonomous decisions based on query patterns. In this evaluation, we used two settings; namely: 1 sensor (contributing 500,000 data points that are combined in a single stream) and 50 parallel sensor streams (each constituting a stream of 10,000 data points). This evaluation setting will impact the performance of the system, as different data stores are maintained per stream. The IoT devices (sensors) in this evaluation produce data every 5 min. The user query aims to retrieve average soil moisture over a specific time window. The window was varied from 1-min to 1-month to observe the response time and validate the performance of the smart summarisation approach. The outcome of the evaluations is presented in Figure 8, Figure 9 and Figure 10.

Figure 8 and Figure 9 provide the query response time for the SmartFarmNet platform without the real-time analysis feature. It is evidentthat the query response time is linear and increases as the number of sensor data points/data streams increases. Each query operation involved fetching a large section of data across 50 data streams using semantic matching, i.e., searching the semantic cloud data store (LSM-Light) for a sensor that produces soil moisture data as opposed to a string search (e.g., SQL like a keyword). Figure 10 presents the query response time to retrieve the sensor data with the real-time statistical analysis feature. As can be noticed, the performance of SmartFarmNet with the real-time analysis is two-fold better than without real-time analytics. By taking advantage of the pre-compute stream summaries that are updated when a new data point arrives or when the time window changes, SmartFarmNet is capable of providing near real-time query response. For complex queries (such as data from multiple days), different micro summaries, such as hourly, daily, weekly, and monthly are used for quick computation. For example, to compute the average for the last four hours and thirty minutes, the hourly average for 4-h along with the raw data obtained for the last 30-min is used. The results show that the increasing velocity of sensor data streams has little or no impact on the performance of the system. This is a key feature of the SmartFarmNet system, making it more scalable than current IoT platforms. This evaluation also demonstrates the suitability of semantic web technologies for storing, analysing, and visualising internet-scale IoT data.

### 6.2. Evaluation of Micro Summary Computation

SmartFarmNet’s real-time analysis feature depends on maintaining an updated micro summary for incoming sensor data streams. In order to estimate the performance of this process, we calculated the computation time to manage and sustain the data structure that keeps track of stream summaries for the 500,000 sensor data points (from 50 sensors) used in the evaluation. The computational process to maintain the data structure is complex, as it requires updating of the stream summaries across two dimensions (namely, each time-window and each stream) continuously. In this evaluation, we aim to establish the maximum time required for summary computation using existing data. Hence, in this evaluation, we assess the SmartFarmNet platform on the task of generating all summaries for the 500,000 data points from 50 sensor streams. Particularly in this assessment, we calculate the total time required to compute summaries for different window sizes. We used the same hardware configuration and sensor sensing frequency as in the previous evaluation. Table 3 presents the outcome of this assessment. As indicated by the results, the maximum time to compute the summary is 25 s (for computing monthly summaries). However, in reality, the summaries are calculated incrementally (i.e., as new data is available in the platform, the summaries are updated). This result indicates the maximum time it could take to recompute the summary in case of system failure. As presented in Section 5.1.1, the raw data from each sensor stream is always persisted to storage, hence reducing the chance of data loss.

## 7. Lessons Learnt

To a great extent, the platform design addressed the unique challenges in building commercial scale IoT systems, identified in Section 3. Below is a summary of our experience and lessons learnt from building a system for smart farming.

**Support for virtually any IoT device:** The key challenge that we faced in developing the SmartFarmNet platform was managing the plethora of Internet of Things devices, ranging from wireless sensor networks to mobile smart phones to cameras, etc. Our solution was to focus on developing common interfaces (API) and consistent representation of sensors and their data using semantic web technologies and thus moving away from the traditional packaged hardware/software solutions. By using a consistent way of representing data and providing different means to ingest data into the system (from API to wrappers built in Java, Python, R, etc.), we were able to interoperate between IoT hardware silos.
**Provide rapid analysis of data in real-time:** One fundamental challenge that underpinned most IoT platforms was in their ability to perform fast analysis of data over a large number of sensor data streams. By employing real-time statistical data analysis, the SmartFarmNet platform was able to achieve this objective. The platform incorporates a scalable methodology, delivering near real-time query response time as compared to traditional SQL-based systems [4].
  **Integration with Semantic Web:** By using the semantic web technologies such as ontologies and linked open data, we were able to 1)use currently available semantic web standards, allowing the system to exchange data with other IoT services in the internet; and 2) enforce standards in IoT application development that is currently not prevalent in most existing solutions. Using the semantic web technologies also enabled the platform to be easily extended to new domains, such as aquaculture, cotton growing, etc. In certain cases, SmartFarmNet was also used as a diagnostic tool to understand the performance of the underlying sensor network and detect failures using a pre-computed error estimate for each sensor.
**Do-it-yourself approach for visualisation and analysis of data:** The design objective of SmartFarmNet was to empower its users by providing standard tools combined with a flexible and powerful API. By employing a do-it-yourself approach, SmartFarmNet has reached a wider set of users and enabled them to collect crop performance data with any sensor(s). The ability for a farmer to explore and analyse crop growth data using simple selection based on familiar concepts such as crop phenotype, treatment, etc., was very useful in breaking many barriers to the SmartFarmNet uptake.

## 8. Conclusions

In this paper, we presented SmartFarmNet, a pioneering effort in building a scalable sensor data acquisition, analysis, and visualisation platform for smart farming applications, based on the Internet of Things. We presented the architectural design of the platform that aims to support virtually any IoT devices, allow rapid ingestion and visualisation of IoT data using zero-programming effort (do-it-yourself principles), and provide a virtual laboratory environment for visualisation and sharing of study data. The proposed SmartFarmNet uses a unique and novel real-time statistical analysis approach that enables near real-time responses to user queries (validating the platform’s ability to scale in order to handle high-velocity data streams). Through evaluation using actual farming data, we validated the elasticity and scalability of the platform.

## Figures and Tables

**Table 1:** Comparison of SmartFarmNet with other Internet of Things (IoT) platforms. EC2: Elastic Cloud Computing.

Platform	Sensor Discovery	Bring-Your-Own IoT Device	Scalable Data Analysis	Sharing Sensor, Data, and Analysis Results (Virtual Lab)
UBIDOTS	Not Supported	Yes, but requires considerable efforts to develop new interfaces	No	No. Only provides API for raw data access
Xively	Partial support with no specific approach for metadata description/management	Yes, but requires considerable efforts to develop new interfaces	No	No. Only provides API for raw data access
SensorCloud	Not Supported	Supports only vendor-specific sensors (some support for CSV file data)	Partial	Partial
IBM Bluemix	Not Supported	Yes, but requires considerable efforts to develop new interfaces	Partial with additional development required	Partial by using existing bluemix infrastructure as a service platform
Amazon IoT	Not Supported	Yes, but requires considerable efforts to develop new interfaces	Partial, with additional development required	Partial by using existing EC2 infrastructure as a service platform
IoTCloud	Not Supported	Yes, but requires considerable efforts to develop new interfaces	No	No
Apache Storm	Not Supported	Yes, but requires considerable efforts to develop new interfaces	No	No
SmartFarmNet	Supported via Semantic Web Technologies	Yes with in-built support for 30+ commercial and experimental sensors	Yes, real-time data analytics functions are built in	Yes, easy to use e-commerce-like use interaction model

**Table 2:** SmartFarmNet platform—implementation details. LSM-Light: linked sensor middleware-light.

Components	Implementation Details
SmartFarmNet gateway (X-GSN)	JAVA-based semantic sensor stream processor. Arduino and ArduCrop sensor wrappers to interface with IoT devices
Cloud Data Store (LSM-Light)	LSM-Light developed using JAVA and Open Virtuoso triple store.
Sensor Explorer	Java applications deployed in JBOSS
Reasoner Service	Apache Jena supported by SmartFarmNet OWL ontology
User Interfaces	Do-it-yourself tools developed in Java Server Faces (JSF)
Data Analytics	Redis [33]

**Table 3:** SmartFarmNet platform—real-time analysis computation time.

Summarisation	Processing Time Total
Hourly	6620 milliseconds
Daily	9971 milliseconds
Weekly	10,926 milliseconds
Monthly	24,543 milliseconds
